# Biophysically Inspired Rational Design of Structured Chimeric Substrates for DNAzyme Cascade Engineering

**DOI:** 10.1371/journal.pone.0110986

**Published:** 2014-10-27

**Authors:** Matthew R. Lakin, Carl W. Brown, Eli K. Horwitz, M. Leigh Fanning, Hannah E. West, Darko Stefanovic, Steven W. Graves

**Affiliations:** 1 Department of Computer Science, University of New Mexico, Albuquerque, New Mexico, United States of America; 2 Center for Biomedical Engineering, University of New Mexico, Albuquerque, New Mexico, United States of America; 3 Department of Chemical and Biological Engineering, University of New Mexico, Albuquerque, New Mexico, United States of America; Tata Institute of Fundamental Research, India

## Abstract

The development of large-scale molecular computational networks is a promising approach to implementing logical decision making at the nanoscale, analogous to cellular signaling and regulatory cascades. DNA strands with catalytic activity (DNAzymes) are one means of systematically constructing molecular computation networks with inherent signal amplification. Linking multiple DNAzymes into a computational circuit requires the design of substrate molecules that allow a signal to be passed from one DNAzyme to another through programmed biochemical interactions. In this paper, we chronicle an iterative design process guided by biophysical and kinetic constraints on the desired reaction pathways and use the resulting substrate design to implement heterogeneous DNAzyme signaling cascades. A key aspect of our design process is the use of secondary structure in the substrate molecule to sequester a downstream effector sequence prior to cleavage by an upstream DNAzyme. Our goal was to develop a concrete substrate molecule design to achieve efficient signal propagation with maximal activation and minimal leakage. We have previously employed the resulting design to develop high-performance DNAzyme-based signaling systems with applications in pathogen detection and autonomous theranostics.

## Introduction

DNA is a versatile nanoscale engineering material. The sequence-specific nature of DNA hybridization via Watson-Crick complementarity and the predictability of DNA secondary structure [1]–[3] make it feasible to rationally design DNA nanostructures [4]–[10], synthetic molecular motors [11]–[16], and dynamic nanoscale logic devices [17]–[24]. Rational design is an important concept because the ability to directly apply biophysical principles [25] and straightforward DNA interaction rules [26], [27] to molecular design is a key reason for the success of DNA nanotechnology. In contrast, recent efforts in metabolic engineering [28] and protein-based molecular computation [29]–[32] show promise because of the wide-ranging chemical repertoire of protein chemistry, but the design process is complicated by the complexity of protein structures and the promiscuous nature of amino acid interactions. This paper concerns the application of biophysical principles in the design of structured nucleic acid molecules to implement molecular logic circuits with DNAzymes. This molecular logic architecture can perform nanoscale computations in response to chemical stimuli, with potential applications in pathogen detection and autonomous theranostic devices.

DNAzymes [33], [34] (also known as deoxyribozymes) are single DNA strands that have been found to catalyze a range of chemical reactions [35]–[48]. The use of DNAzymes for molecular logic is well reported in the scientific literature [49]–[59]. RNA-cleaving DNAzymes are the most widely used and best characterized, owing to their potential for therapeutic applications [59], [60]. We have previously reported [61] molecular logic gates based on regulating the 8–17 DNAzyme [45], [62], [63] by toehold-mediated strand displacement (TMSD) reactions [21], which provide a precise means of controlling DNAzyme activation. The 8–17 DNAzyme can cleave a chimeric DNA-RNA substrate at a cleavage site denoted by a single RNA base. We use the 8–17 DNAzyme here because of its compact size and high turnover rate [64]. We refer to these as *DNAzyme displacement* (DzD) logic gates.

Connecting multiple DNAzyme logic gates into signaling circuits is necessary to increase their computational power beyond that of parallel DNAzyme gate arrays [50], [51], [54], [55] and to incorporate non-trivial circuit motifs analogous to cellular signaling and regulatory cascades, *e.g.*, as feedback cycles. For multiple RNA-cleaving DNAzyme logic gates to interact, the activity of one DNAzyme (referred to as the upstream DNAzyme) must modulate the activity of a second (downstream) DNAzyme. Here we achieve this by designing a substrate molecule that interacts with the downstream DNAzyme only after being cleaved by the upstream DNAzyme. This is a non-trivial engineering challenge because the shorter, cleaved product must readily interact with the downstream DNAzyme, whereas the longer, pre-cleaved substrate (which contains the product as a subsequence) must not interact with the downstream DNAzyme. Previous attempts to address this problem have either required micromolar input concentrations [56] or additional structures that increase circuit complexity [65]–[67], or they were unable to further propagate a logic signal to downstream circuit elements [68], [69]. These restrictions limit their practical applicability.

We have developed a solution to this problem via a structured chimeric substrate (SCS) molecule that connects two DzD logic gates by acting as a signaling intermediary [70]. The SCS is a single-stranded molecule that sequesters a downstream effector sequence within its secondary structure. Upon cleavage by an upstream DNAzyme, the structure undergoes a conformational change and frees up the effector sequence to interact with the downstream DNAzyme. This signaling interaction is a complex, multi-step process with many reactions whose rates must be appropriately balanced to achieve acceptable performance. In this paper we describe an iterative, rational design approach that is suitable for developing any structured DNA molecule, but is of particular value in the development of the SCS design for the construction of multi-layer DzD signaling cascades and logic circuits. We used this approach to translate biophysical insights into an efficient, modular SCS design.

## Materials and Methods

### Sequence design

We fixed a downstream DNAzyme sequence based on our previous work [61] and used NUPACK [71], [72] in conjunction with the Pyxis framework [73] to perform structural modeling using the ISO representation of nucleic acid secondary structure [74]. We used this software to encode the desired SCS structures and search for candidate sequences for the SCS molecule and corresponding upstream DNAzyme. In the sequence search procedure, we included both the uncleaved SCS structure (with the designed secondary structure specified) and the cleavage product that serves as a downstream activator strand (ACT, with no secondary structure specified) to ensure that both folded properly. We calculated activation and leakage scores for each candidate design as the percentage of binding between the SCS or ACT to the downstream inhibitor at thermodynamic equilibrium in the presence of the downstream DNAzyme. A well-protected SCS structure is one for which minimal interaction is predicted with the downstream inhibitor in the presence of the downstream DNAzyme, suggesting that the downstream DNAzyme-inhibitor complex is more thermodynamically favorable than spurious downstream DNAzyme activation. Similarly, an efficient ACT structure is one for which significant binding is predicted with the downstream inhibitor in the presence of the downstream DNAzyme, suggesting that activation of the downstream DNAzyme is more thermodynamically favorable than preserving the catalytically inactive downstream DNAzyme-inhibitor complex [4]. We quantified these effects as the percentage of downstream DNAzyme released (as opposed to bound to the downstream inhibitor) in both cases. In our experience, good SCS structures yielded <1% DNAzyme release, while good ACT candidates yielded roughly 40-60% DNAzyme release, and excellent ACT candidates yielded around 60–80% DNAzyme release.

### Materials

All oligonucleotides were purchased from Integrated DNA Technologies (Coralville, IA). Oligonucleotides were purchased with standard desalting where possible; DNA-RNA chimeric substrate molecules (SCS molecules and linear reporter substrates) were purified using RNAse-free HPLC. Sequences for all oligonucleotides used herein are presented in Table 1. Oligonucleotides purified using standard desalting were resuspended in RNAse-free H_2_O (Sigma-Aldrich, St. Louis, MO) at a stock concentration of 50 µM. These stocks were diluted to working stocks of 2.5 µM, by diluting 50 µL stock DNA into 950 µL assay buffer (described below). RNAse-free HPLC oligonucleotides were resuspended directly at 2.5 µM in RNAse-free H_2_O.

**Table 1:** Oligonucleotide sequences, listed from 5′ to 3′.

Design	Strand	Sequence
Fixed	Dz2	GAACTATCTCCGAGCCGGTCGAAAACTAAGA
Fixed	Sub	FAM-TCTTAGTT**rAG**GATAGTTCAT-TAM
1	Inh2	CTCCATCTTAGTTTTCGACCGGCT
1	SCS	CTCCATCTTAGTTTTCGGGTATT**rAG**GCGGACAGCCGGTCGAAAACTAAGATGGAG
1	Dz	GTCCGCTCCGAGCCGGTCGAAAATACCC
2	Inh2	CTCCATCTTAGTTTTCGACCGGCT
2	SCS	AGCCGGTCGAAAACTAAGACGTGAGGGTATT**rAG**GCGGACTCACG
2	Dz	GAGTCCGCTCCGAGCCGGTCGAAAATACCCT
3	Inh2	CGGGTTCTTAGTTTTCGACC
3	SCS	AGCCGGTCGAAAACTAAGACGCCCAGGGTATT**rAG**GCGGACTGGGCG
3	Dz	GTTTATGCTCCGAGCCGGTCGAAACCCGTTTCT
4	Inh2	GAAGTTCTTAGTTTTCGACC
4	SCS	GGGATGTGAAGT**rAG**GATGGGACGGTCGAAAACTAAGAACTTCAC
4	Dz	GTCCCATCTCCGAGCCGGTCGAAACTTCACATCCC
5	Inh2	CGTATTCTTAGTTTTCGACC
5	SCS	GGTCGAAAACTAAGAATACGGGACTACAGTTAGTAGT**rAG**CGTATGAGGG
5	Dz	CCCTCATACGCTCCGAGCCGGTCGAAACTACTAACT
6	Inh2	GTAGCTCTTAGTTTTCGACC
6	SCS	CACGCGTAGCGGTCGAAAACTAAGAGCTACAAT**rAG**GCGTGAGG
6	Dz	CCTCACGCTCCGAGCCGGTCGAAATTGTAGC
7	Inh2	ATGTATCTTAGTTTTCGACC
7	SCS	CACGCCTATCTTAGGTCGAAAACTAAGATTCATTTACT**rAG**GGCGTGATTAG
7	ACT	CACGCCTATCTTAGGTCGAAAACTAAGATTCATTTACTA
7	Dz (11/10)	CTAATCACGCCTCCGAGCCGGTCGAAAGTAAATGAA
7	Dz (11/8)	CTAATCACGCCTCCGAGCCGGTCGAAAGTAAATG
7	Dz (10/8)	TAATCACGCCTCCGAGCCGGTCGAAAGTAAATG
8	Inh2	ATGTATCTTAGTTTTCGACCGGC
8	SCS	CGCCCTAATCTTAGGTCGAAAACTAAGATACATACT**rAG**GGCGTGATG
8	Dz	ATCACGCCTCCGAGCCGGTCGAAAGTATGTA
8 (Fig 7)	SCS	CGCCACAATCTTAGGTCGAAAACTAAGATACATACT**rGU**GGCGTGATG
8 (Fig 7)	Dz	ATCACGCCAGGCTAGCTACAACGAAGTATGTA

Dz  =  upstream DNAzyme, SCS  =  structured chimeric substrate, ACT  =  downstream activator (one SCS cleavage product), Inh2  =  downstream inhibitor, Dz2  =  downstream DNAzyme, Sub  =  downstream readout substrate. FAM  =  fluorescein, TAM  =  TAMRA. The ribose adenine and guanine bases in cleavable SCS molecules are denoted by rA and rG respectively, and the cleaved dinucleotide junctions are picked out in boldface. Strands whose sequences are fixed across all designs are annotated as “fixed”. For Design 7, the Dz variants are also annotated with the lengths of their 5′/3′ substrate binding arms.

### Gate preparation

Typically, 60 µL of DNAzyme solution and 75 µL of inhibitor solution, taken from 2.5 µM working stock solutions, were combined and heated at 95°C for 3 minutes on a heat block and subsequently annealed by cooling to room temperature over a minimum of 90 minutes. This produced a solution with 25% excess inhibitor free in solution. All other species that required an initially hybridized state (SCS and activator molecules) were prepared using the same annealing protocol.

### Assay conditions and instrumentation

All assays were performed at room temperature (∼23°C) in a buffer of 1 M NaCl, 50 mM HEPES, 1 mM ZnCl_2_, pH 7.0, with the exception of the experiment with the upstream 10–23 DNAzyme, which was performed in a buffer of 1 M NaCl, 50 mM HEPES, 20 mM MnCl_2_, pH 7.47. Species were added in the following order and in the specified concentrations: substrate (50 nM), downstream DNAzyme-inhibitor complex (100 nM with 20 nM excess inhibitor), and SCS or downstream activator depending on the experiment (100 nM). The upstream DNAzyme (100 nM) was added last to initiate cleavage where required. Fluorescence was read on either a Spectramax M2e fluorescent plate reader (Molecular Devices, Sunnyvale, CA) in a 200 µL reaction volume or a Quantamaster 40 fluorimeter (PTI, Binghamton, NJ) in a 300 µL reaction volume. Fluorescence was monitored at 492 nm excitation and 518 nm emission wavelengths. Each kinetic trace is representative of multiple experiments run with each particular SCS design.

## Results

### Design Criteria for DNAzyme Signaling Cascades

**Figure 1:**
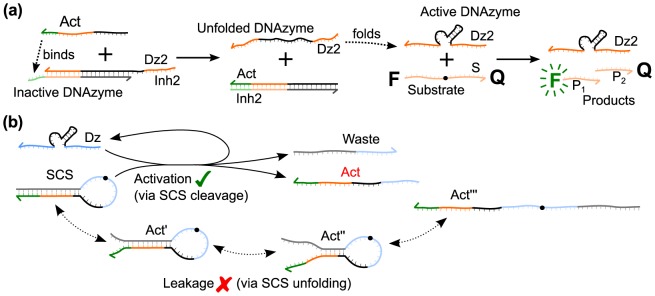
DNAzyme displacement and DNAzyme cascade mechanisms. (a) DNAzyme displacement mechanism. The DNAzyme (Dz2) is initially inactivated by pre-hybridization to an inhibitor strand (Inh2) in the Dz2-Inh2 complex. Once released, the activator binds to the complementary inhibitor toehold, displacing Dz2 and producing an inert waste complex (Act-Inh2). The displaced Dz2 strand can now fold into a catalytically active conformation and proceed to cleave its substrate. In this paper we consider only two-layer cascades in which Dz2 cleaves a substrate labeled with a fluorophore and a quencher to generate a fluorescent readout by loss of FRET. (b) Cartoon depicting the high-level pathways of DNAzyme cascade operation using a simple hairpin-based SCS design (see Figure 3). The desired pathway is labeled “activation”, in which the upstream DNAzyme (Dz) catalyzes the cleavage of the SCS molecule to release a downstream activator strand (Act) from its sequestered state, which can initiate a downstream DNAzyme displacement reaction, as illustrated in part (a). The undesired pathway is labeled “leakage”, in which instabilities in the SCS structure spontaneously reveals the activator sequence which can occur due to sequence impurities, structural isoforms, and natural stochastic fluctuations. A hypothetical leakage pathway is shown which produces a series of downstream activators (Act', Act”, Act’’’). These structures may refold into the initial SCS structure or may initiate a downstream DNAzyme displacement reaction, thereby activating downstream DNAzymes in the absence of active upstream DNAzymes. The goal of the rational design process described in this paper was to develop a concrete design for the SCS molecule that achieves maximal activation with minimal leakage.

Our objective was to create a design process by which DNAzyme-based logic gates could be scaled into complex decision networks that were suitable for biodetection applications: this required circuits to operate at near equimolar component concentrations and be amenable to low (pM to nM) input and gate concentrations. The latter constraint precluded relying on concentration effects to bias competitive binding interactions. Therefore, to avoid competition we introduced an intermediary molecule capable of transmitting information between one DNAzyme and another. The DzD mechanism of regulating DNAzyme catalysis using TMSD reactions [61] is illustrated in Figure 1a. The DzD mechanism allows rational programming of reaction pathways by kinetic and thermodynamic means, and we have found that this approach for regulating DNAzyme catalysis is particularly well suited for use in signaling cascades [70].

### SCS Design Strategy

The choice of DNAzyme displacement to regulate catalysis suggested a strategy for implementing DNAzyme signaling: to sequester the downstream activator (ACT) strand. In particular, sequestering the downstream activator toehold in the secondary structure of an intermediary substrate molecule should prevent it from interacting with the downstream DNAzyme. Then, subsequent binding and cleavage of the intermediary by an upstream DNAzyme will cause a structural change that releases the activator sequence so that it can interact with the downstream gate. In this manner, an activation signal is passed from the upstream DNAzyme to the downstream DNAzyme. Because we use substrate molecules consisting of both DNA and RNA bases, we call this intermediary a structured chimeric substrate (SCS).

Figure 1b shows a schematic of desired and undesired SCS interaction pathways in a DNAzyme signaling cascade using a basic SCS design for illustrative purposes. In the “activation” pathway from Figure 1b, the active upstream DNAzyme (Dz) cleaves the SCS to release a downstream ACT strand. This may be a complex, multi-step process initiated via a binding step between the upstream DNAzyme and the SCS. The efficiency of the binding reaction depends on the structure of the SCS and the corresponding binding pathway for the upstream DNAzyme. When the DNAzyme is stably bound, it must hydrolyze the RNA base to cleave the SCS. The rate of this cleavage reaction is affected by many factors, such as the type of DNAzyme, buffer conditions, and conformational fluctuations within the DNAzyme-SCS complex. After cleavage, the DNAzyme must then dissociate from the products at a rate dependent on the length of the DNAzyme binding arms. For binding arms of 8 nucleotides or less, product rebinding after dissociation has a negligible effect on DNAzyme kinetics due to the low melting temperature of an 8 bp duplex.

Following activation, the activator released from the SCS structure by the cleavage reaction interacts with the inactive downstream DNAzyme (Dz2-Inh2 complex) to release an active downstream DNAzyme (Dz2) via a DzD reaction, as illustrated in Figure 1a. The rate of this reaction will depend on any secondary structure in the activator released by SCS cleavage and the toehold lengths in the ACT strand and in the Dz2-Inh2 complex. The activated Dz2 DNAzyme can cleave multiple FRET-labeled reporter substrates for an amplified fluorescent readout, or alternatively cleave other SCS molecules for further signal propagation.

The “leakage” pathway from Figure 1b accounts for non-specific activation of the downstream DNAzyme through interactions with uncleaved SCS molecules. Leak reactions may be caused by incomplete sequestration of the downstream activator or by fluctuations and imperfections in the SCS structure. This may partially or completely reveal the downstream activator, enabling the uncleaved SCS to bind directly to the Dz2-Inh2 complex and spuriously activate the downstream Dz2 DNAzyme. Although steps in the leakage reaction may correspond to steps in the activation reaction, the lack of SCS cleavage means the entire SCS strand remains intact during the process. This will lead to a different rate constant for the leakage reaction than for activation, and the leakage mechanism may be different. Although binding to the toehold is the most likely leakage mechanism, invasion of the Dz2-Inh2 complex via the catalytic core due to DNA breathing may also occur.

The rates of the activation and leakage pathways derive from the structure of the SCS molecule based on kinetic and thermodynamic considerations, *e.g.*, the relative thermodynamic favorability of the hybridization between the SCS or ACT with the downstream DNAzyme inhibitor. These parameters can be balanced by using biophysical principles to predict the effects of design changes on these rates. Before SCS cleavage, the retention of the secondary structure of the SCS via intramolecular interactions should be thermodynamically favorable. After cleavage, the interaction of the SCS product and the downstream inhibitor should be thermodynamically favorable. Therefore, the SCS structure must be designed to balance the thermodynamic stability of the pre-cleaved state (to minimize leakage) with that of the post-cleavage state (to maximize activation).

Hence, the design objectives for the rational design signaling intermediaries to implement DNAzyme signaling cascades can be summarized as follows:

1. Efficient binding and cleavage of the SCS by the upstream DNAzyme.
2. Efficient activation of the downstream DNAzyme by the activator released by SCS cleavage.
3. Robust sequestration of the downstream activator in the pre-cleavage SCS structure.

**Figure 2:**
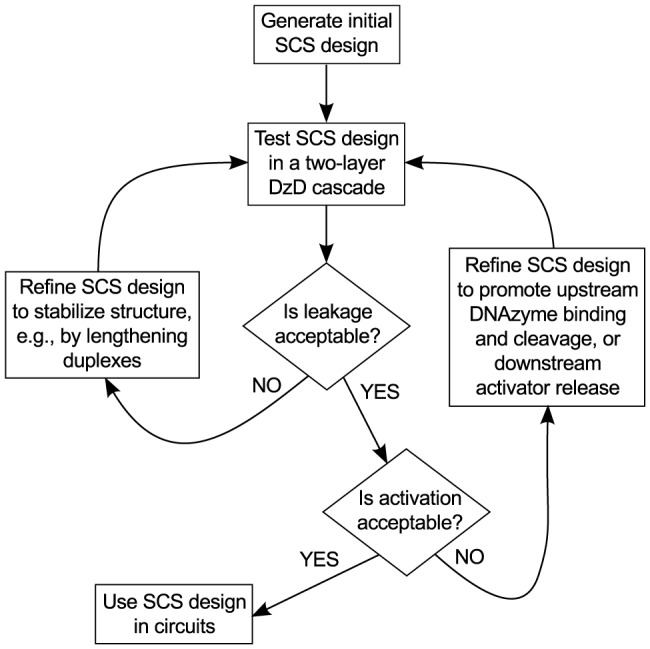
Flowchart of the iterative design process. Design testing and refinement are integrated in a feedback process that used biophysical insights to reduce leakage or promote activation, as appropriate. In the feedback cycle where leakage is unacceptable, additional stabilization of the structure is required to prevent spontaneous downstream activation by uncleaved SCS molecules. There are a number of possible remedies, and it may take several experiments to identify which step is causing the problem.

An iterative design process to achieve these objectives is presented in Figure 2. In the remainder of this section, we detail the engineering design process that we employed to obtain a structure that satisfies these constraints.

### SCS Design 1

**Figure 3:**
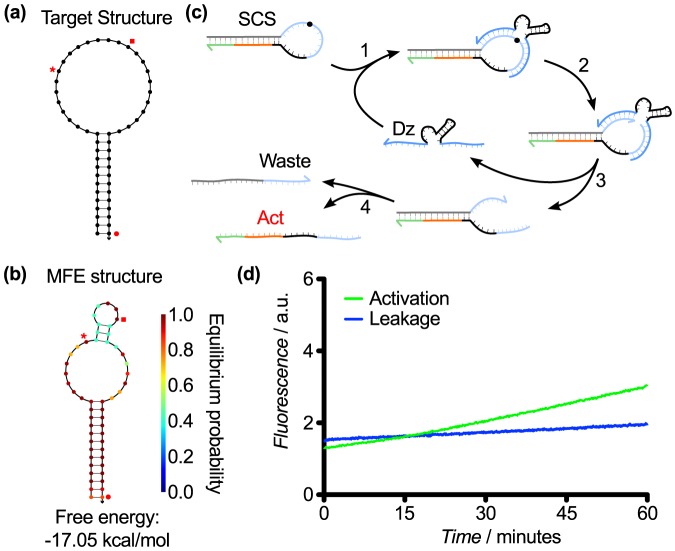
SCS Design 1. (a) Target structure. In this and subsequent Figures, the red disc denotes the beginning of the activator toehold, and the end of the activator sequence is denoted by the red square. The red star marks the cleavage site. (b) MFE structure of the SCS Design 1 sequence from Table 1. (c) Hypothesized mechanism for cleavage of Design 1, resulting in the release of an activator (Act) that can instigate a downstream TMSD reaction. (d) Response of Design 1 over 60 min. Leakage (downstream activity in the absence of the upstream DNAzyme) was negligible; however, activation (downstream activity in the presence of the upstream DNAzyme) was very slow. Here and henceforth, “a.u.” denotes “arbitrary units” for fluorescence measurements.

Design 1 used a basic stem loop structure with a 26 nucleotide (nt) loop and a 13 nt stem. In our early design iterations, the sequestered activator sequence was 24 bases long, made up of a 5 nt toehold, an 8 nt substrate binding arm, and an 11 nt core sequence. This meant that the toehold, along with a significant portion of the activator, was sequestered in the stem (Figure 3a,b). The remainder of the activator continued into the loop, which also contained the cleavage site and substrate binding arms of the upstream DNAzyme. We hypothesized that the upstream DNAzyme would bind to the loop and cleave the RNA base in the loop, which would split the stem loop into two strands, allowing them to dissociate (Figure 3c). Once the strands unbound, the toehold of the activator would be free to displace an active DNAzyme from the downstream Dz2-Inh2 complex, as shown in Figure 1a. Testing of Design 1 resulted in very low leakage but almost no activation (Figure 3d). The fact that some activation was observed indicated that some cleavage of the SCS structure was taking place. We surmised that the low activation was due to low product dissociation caused by the high stability of the relatively long stem, which would prevent the activator toehold from binding to the downstream gate.

### SCS Design 2

**Figure 4:**
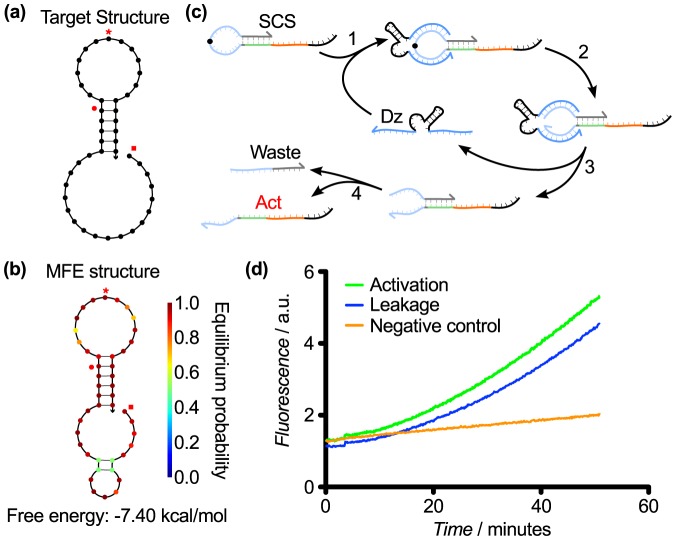
SCS Design 2. (a) Target structure. (b) MFE structure of the SCS Design 2 sequence from Table 1. (c) Hypothesized mechanism for cleavage of Design 2, resulting in the release of an activator (Act) that can instigate a downstream TMSD reaction. (d) Response of Design 1 over 60 min. The shorter stem decreased SCS stability, resulting in increased leakage; and the rate of activation compared with leakage was negligible, likely due to the inefficiency of catalyzing hydrolysis of a cleavage site in a loop. The negative control is the downstream activity in the absence of both the SCS and the upstream DNAzyme.

Design 2 tested this hypothesis by shortening the stem loop to 5 base pairs. The activator was tested in a reverse orientation in SCS structure; the enzyme binding arm and core sequence of the activator was left single-stranded, extending from the 5′ side of the SCS while the toehold remained bound in the stem (Figure 4a,b). In this and subsequent iterations, we used optimized downstream DzD gates in which the length of the inhibitor strand was reduced to 20 bases, which allowed us to correspondingly shorten the activator, removing 4 bases from the core displacement sequence. This enabled us to design more compact SCS structures, which we expected would increase activator sequestration. Since the complementary sequence on the inhibitor is normally hybridized with the downstream DNAzyme, we hypothesized that having the activator single stranded for these domains would not result in significant activation because toehold binding is still required to initiate the reaction (Figure 4c). Testing of Design 2 showed moderately increased activation but almost as high leakage compared to Design 1, presumably both due to the shortened stem. Overall, however, this design iteration did not significantly improve the cascade signal (Figure 4d).

### SCS Design 3

**Figure 5:**
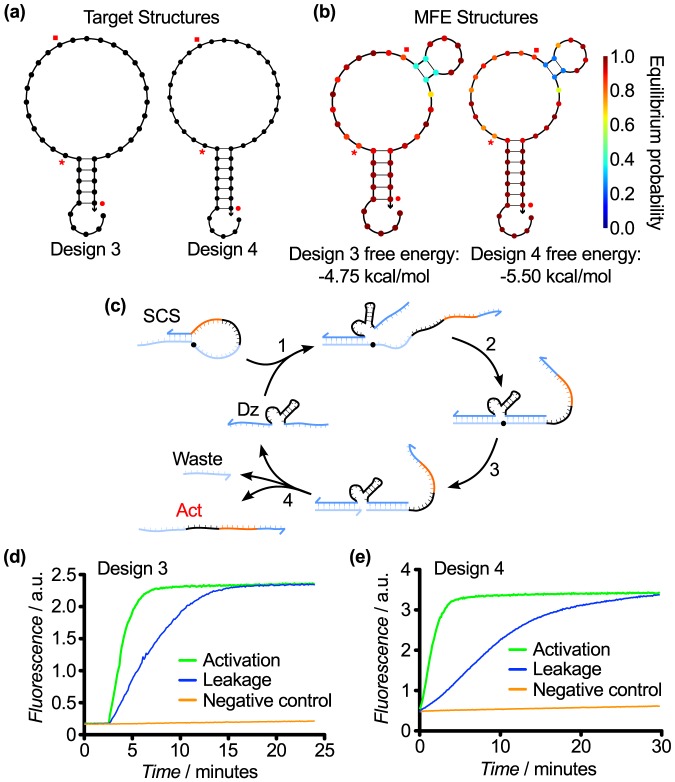
SCS Designs 3 and 4. (a) Target structures, which vary in the stem length. (b) MFE structures of the SCS Design 3 and 4 sequences from Table 1. (c) Hypothesized mechanism for cleavage of Designs 3 and 4, illustrated with Design 3. (d) Response of Design 3 over 25 min. (e) Response of Design 4 over 30 min. For both designs, rapid activation was achieved. However, the rate of leakage also increased, indicating that the protection of the toehold was insufficient. This was likely due to the relatively short stem and large loop. The leakage was lower with Design 4 due to the longer stem.

For the third design iteration, we refocused our efforts on the location of the cleavage site. Designs 1 and 2 placed the cleavage site in the loop, which required the DNAzyme to bind to a structured substrate, as opposed to the standard linear substrate molecule. Furthermore, the efficiency of the DNAzyme-catalyzed RNA hydrolysis reaction requires the DNAzyme to position the RNA base in a specific conformation [75]. We hypothesized that the torsional strain of the loop likely slowed down the binding and cleavage reactions by preventing efficient formation of duplexes between the DNAzyme and SCS and by reducing the DNAzyme's ability to properly orient the RNA base, respectively. Therefore, for Design 3 we redesigned the SCS structure to favor more efficient upstream binding and cleavage (Figure 5a,b). We hypothesized that the 3′ binding arm of the upstream DNAzyme would hybridize to the 5′ toehold extending from the SCS stem. The substrate binding arm would then act as an invader strand in a TMSD reaction, displacing the stem and opening the loop. The second binding arm of the upstream DNAzyme could then bind to its complementary sequence in the loop, creating a linear substrate properly oriented for efficient cleavage of the RNA base (Figure 5c).

However, Design 3 introduced some sequence constraints between the upstream and downstream DzD gates because the stem sequence was now expected to perform two functions: 1) to protect the downstream toehold, and 2) to serve as the binding arm displacement sequence for the upstream enzyme. Thus, the upstream DNAzyme and activator toehold had a common subsequence, which restricted the design of DNAzymes that may interact using this SCS structure. We considered this to be an acceptable restriction because the downstream toeholds were still free to vary (provided they are free of secondary structure) and because any unwanted hybridization between the upstream DNAzyme and the downstream toehold would be transient due to the short (5nt) length of the complementary sequence.

Testing of Design 3 resulted in an extremely rapid activation rate but also a significant leakage rate (Figure 5d). This suggested that our attempts to promote efficient cleavage were successful. However, the high rate of leakage indicated this SCS structure design was not stable enough to prevent the activator from interacting with the downstream toehold prior to SCS cleavage. We surmised that this was influenced both by the length of the stem and the size of the loop: a short (5 nt) stem is likely to be significantly destabilized by DNA breathing, and a large (25 nt) loop may make it difficult for the SCS to regain the intended structure if the stem does unbind. This hypothesis is consistent with previous work [76] which has shown that the rate of stem closure is strongly dependent on loop size: bigger loops close more slowly than smaller loops. This has important implications as large stem loops that spontaneously dissociate are likely to remain in an open conformation for a significantly longer time, increasing the probability of unwanted interactions with the downstream DNAzyme-inhibitor complex.

### SCS Design 4

Design 4 was a refinement of Design 3 in which the stem was extended to 7 nt but the cleavage site was kept in the same position in the loop, with the goal of maintaining rapid activation while reducing leakage (Figure 5a,b). The hypothesized mechanism was the same as for Design 3 (Figure 5c), and testing revealed that leakage was, indeed, reduced (Figure 5e). However, the extended stem required the use of correspondingly extended substrate binding arms in the upstream DNAzyme so that the binding DNAzyme could displace the entire stem to linearize the SCS molecule. The effects of DNAzyme binding arm length on catalytic activity have been well characterized [77], and the optimal length for rapid product dissociation has been determined to be 8 nt. Hence, the constraints on binding arm length imposed an additional constraint on SCS design that prevented us from arbitrarily extending the stems to stabilize the structure. Respecting the 8 nt limit on substrate binding arm length ensured that binding, cleavage, and product dissociation could all occur efficiently, essential to achieve multiple turnover in the cleavage reaction, the main advantage of using DNAzymes for such reactions.

### SCS Design 5

**Figure 6:**
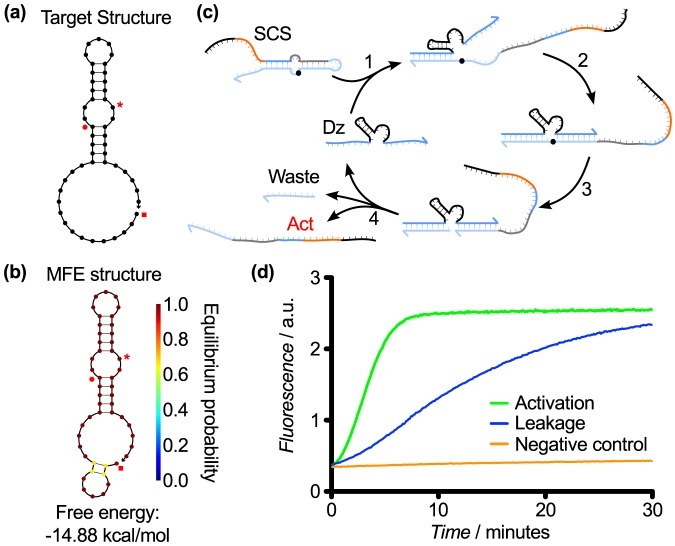
SCS Design 5. (a) Target structure. (b) MFE structure of the SCS Design 5 sequence from Table 1. (c) Hypothesized mechanism for cleavage of Designs 3 and 5. (d) Response of Design 5 over 30 min. This design displayed similar activation and leakage to Design 4.

Since Designs 1 and 2 excelled at activator sequestration whereas Designs 3 and 4 excelled at downstream activation, we developed a hybrid structure for Design 5 that combined the strand displacement and linear substrate alignment mechanism of Design 3 with the reversed activator orientation of Design 2 (Figure 6a,b). Positioning of the activator as a single-stranded overhang enabled the loop size to be significantly reduced, which increased the rate of stem rebinding after spontaneous dissociation, *e.g.*, due to DNA breathing or thermodynamic effects. The cleavage site was left unhybridized, creating a 2 nt bubble, resulting in a dual stem and loop structure. This enabled us to use 5 nt stems, which are beneficial for rapid activation, and allowed us to add a second short stem to increase overall stability. We surmised that these two changes would preserve the structure through multivalent interactions because the degradation of the structure would only occur after two separate stem dissociation events, the first initiated at the toehold and the second initiated in the inner loop. We hypothesized that only the outer stem would dissociate after cleavage, releasing the toehold domain while allowing the inner stem to refold. This was acceptable since the inner stem does not participate in the downstream interaction. In this design, the 5′ arm of upstream DNAzyme binds to the 3′ toehold of the SCS, initiating displacement of the outer stem. The 3′ arm binds to the inner loop and displaces the inner stem (Figure 6c). Although we hoped to increase stability compared to previous designs, this design showed only moderately improved gate leakage in experimental tests (Figure 6d).

### SCS Design 6

**Figure 7:**
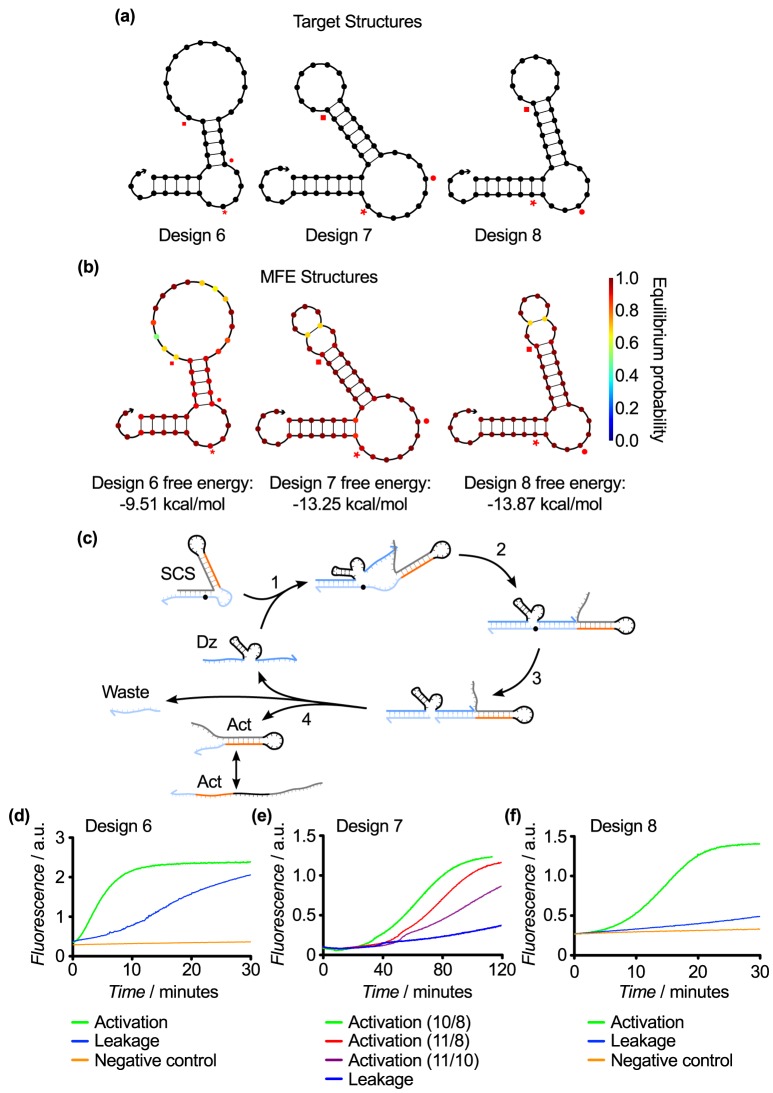
SCS Designs 6, 7, and 8. (a) Target structures, which vary in the stem lengths and loop sizes. (b) MFE structures of the SCS Design 6, 7, and 8 sequences from Table 1. (c) Hypothesized mechanism for cleavage of Designs 6, 7,and 8, illustrated with Design 8. (d) Response of Design 6 over 30 min. This design had a good activation rate and a lower leakage rate compared with the earlier designs, but further optimization was required. (e) Response of Design 7 over 120 min. This design significantly decreased the leakage rate at the expense of activation. We tested various lengths for the substrate binding arms of the upstream DNAzyme (denoted as 5′/3′ in the Figure). These results showed that longer substrate binding arms decrease the effect on activation rate by reducing the rate of product dissociation. (f) Response of Design 8 over 30 min. This design showed a marked improvement in activation rate while retaining a low rate of leakage. The key design change was to shift the cleavage site into the outer stem and keep both loops as short as possible, which allowed an upstream DNAzyme with 8 nt substrate binding arms to be used in conjunction with relatively stable stems.

We retained the dual-stem design motif that we developed for Design 5, in which the activator sequence moved back to the 3′ side of the SCS. This ensured that the activator sequence was now bound back into a loop. In Design 6, each of the stems was 5 nt in length and formed two loops: an inner loop which contained the activator sequence and an outer loop which separated the two stems. The cleavage site was placed in the middle of the outer loop (Figure 7a,b). The upstream substrate binding arm was expected to bind to the toehold on the 3′ side of the stem loop and initiate strand displacement of the outer stem. The other substrate arm was intended to bind the outer loop and displace through the inner stem. Cleavage would render the outer stem as a waste product, while the inner stem containing the toehold would remain intact (Figure 7c). This design relied on the relative instability of the inner stem and loop, so that after the cleavage and dissociation of the outer stem, the inner stem would still activate the downstream gate despite the toehold theoretically being protected in the stem loop. Testing of this gate design revealed that sequestering the activator within the structure, as opposed to placing it in a single-stranded overhang, reduced leakage slightly at the cost of a commensurate reduction in activation rate (Figure 7d).

### SCS Design 7

We next sought to optimize the dual stem-loop design from Design 6 by extending the stems to reduce leakage through increased structural stability. As in Design 6, the cleavage site was located in the outer loop. As in Design 4, the extended stems required the use of upstream DNAzymes with extended substrate binding arms (Figure 7a–c). We examined the response of this SCS molecule to upstream DNAzymes with varying substrate binding arm lengths (Figure 7d). The extended stems in the SCS reduced the leakage significantly compared to previous designs, however extended DNAzyme binding arms were required to achieve significant activation after 120 min (Figure 7e). Since 8 nt substrate binding arms are generally considered to be optimal, we undertook another design iteration to address this issue.

### SCS Design 8 (Final design)

The goal of Design 8 was to retain the basic structure and low leakage of Design 7 while enabling the use of DNAzymes with 8 nt substrate binding arms. A notable innovation was moving the cleavage site into the double-stranded outer stem (Figure 7a–c). This had several effects. First, the position of the cleavage site enabled us to use DNAzymes with 8 nt binding arms that do not displace the entire outer stem and instead rely on spontaneous unbinding of the remaining bases in the outer stem to enable rapid product release. Second, we could minimize the size of the outer loop, which would sequester the downstream toehold more efficiently and reduce leakage. Third, we were able to retain the longer 7 nt stems, which were found in Design 6 to stabilize the structure and give low leakage. Finally, the hybridization of the cleavage site in the outer stem may have also served to protect the RNA base from degradation, which may have contributed to leakage rates. Experimental testing of Design 8 showed that the activation rate was improved while leakage was suppressed, as desired (Figure 7f).

In this design iteration, we also introduced a 3 nt extension of the inhibitor strand that enabled it to bind further into the core while keeping the activator length fixed. Hence, the extra bases on the inhibitor act as a clamp to prevent strand invasion of the downstream DNAzyme-inhibitor complex via the catalytic core. They also act as a short toehold that slows the downstream DzD reaction and enables displaced DNAzymes to rebind to the inhibitor strand in the waste complex. This provides a mechanism to reverse unwanted DNAzyme release via leak reactions. We surmise that this contributed to the reduced leakage. Thus, through eight design iterations, we have developed a stable and reliable SCS structure that facilitates communication in a two-layer DNAzyme signaling cascade. It has a robust activation response in the presence of an upstream DNAzyme and minimal leakage in its absence.

### Demonstration of SCS application in a heterogeneous DNAzyme cascade

**Figure 8:**
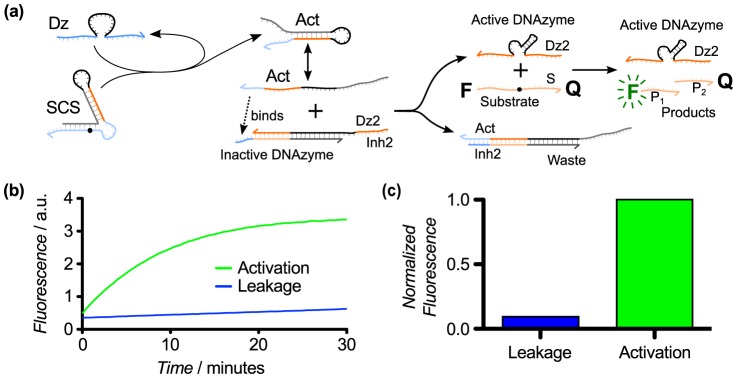
Example application of SCS Design 8 in a heterogeneous two-layer DNAzyme cascade. (a) Cascade schematic for a two-layer cascade, in which the downstream DNAzyme uses the 8–17 catalytic motif (as before), whereas the upstream DNAzyme uses the 10–23 catalytic motif. This cascade uses the final SCS structure (Design 8) shown in Figure 7a,b, with a modified cleavage site sequence to enable efficient cleavage by the upstream 10–23 DNAzyme. (b) Kinetic response of the heterogeneous DNAzyme cascade over 30 minutes. As in our previous experiments using SCS Design 8, we saw strong activation with low leakage. (c) Normalized change in fluorescence plot for the experiment from part (b), showing the gain in fluorescence value measured at t = 30 relative to the leakage value at time t = 0. The data illustrate the broad applicability of our SCS design to the development of signaling cascades between a variety of DNAzymes.

To demonstrate the broader applicability of our SCS design for DNAzyme cascading, we used the final SCS design (Design 8) to implement a two-layer DNAzyme cascade in which an upstream 10–23 DNAzyme [45] activates a downstream 8-17 DNAzyme, as shown in Figure 8a. We retained the SCS Design 8 structure while altering the dinucleotide junction at the cleavage site such that it can be cleaved by an upstream 10–23 DNAzyme. The cascade response in the presence and absence of the upstream DNAzyme (Figure 8b,c) was similar to that previously observed for this SCS design (Figure 7f). The use of different DNAzyme catalytic motifs for the upstream and downstream DNAzymes demonstrates that various DNAzyme motifs can catalyze a variety of chemical reactions [35]–[48] and may be combined in a single system. In other work [70], we have used SCS Design 8 to implement DNAzyme signaling cascades with up to five layers deep by reproducing this structure with different sequences to link different DNAzyme pairs. In that paper, we also implemented two-layer, multi-input DNAzyme logic circuits. These results demonstrate that the SCS design can be composed to design larger DNAzyme circuits.

## Discussion

### Biophysical insights in DNA circuit design

This work demonstrates the rigorous application of biophysical principles to the design of non-trivial DNA secondary structures with specific dynamic behaviors. This approach was made possible by the predictability of DNA secondary structure and by our use of toehold-mediated strand displacement to encode desired interaction pathways into the structure. We were able to adjust the stability of various structural motifs by controlling the lengths of duplexes and to control binding kinetics by adjusting toehold lengths.

Our experience demonstrates that a number of potential design variants for the SCS molecule can be used to create a wide variety of behaviors. We found that structurally similar designs may yield comparable performance via different reaction mechanisms and that minor structural modifications can have significant impacts on the performance. For example, the final few design iterations (Designs 7–9) were variations on a theme with significantly differing performance. Furthermore, although we discarded the intermediate designs described in this paper because they failed to meet our performance criteria in conjunction with downstream DzD gates, it is possible that some of these structures may prove useful in other settings.

### Optimization of testing procedures

While early designs were purchased directly as a DNA structure with RNA cleavage site, necessitating a significant surcharge for RNAse-free purification, later designs were initially tested using a DNA-only form of both the SCS and activator (SCS post-cleavage). This allowed us to assess the hypothetical minimum and maximum circuit response, wherein gate response in the presence of the SCS would be a measure of leakage, and the response in the presence of activator would be the positive response observed if all SCS molecules are cleaved. This was a reasonably good approximation of SCS behavior and helped rule out nonviable structures before they were purchased with the RNA base. That it was both time and cost effective as well was highly beneficial, and this approach significantly sped up the testing time for each new SCS design.

Due to the high cost and time of synthesis of the linear FRET substrate for fluorescent readout of downstream DNAzyme activity, we fixed the downstream reporter layer and designed various upstream layers using different SCS molecules. This imposed constraints on SCS sequences, meaning that some designs resulted in more viable SCS sequence candidates than others. While the optimized design of an entire cascade would be highly desirable, this comes at a significant computational and material cost because the number of potential designs increases if the sequences of multiple layers are optimized simultaneously during the design process.

### Limitations of thermodynamic modeling for cascade design

Thermodynamic modeling was used to guide optimization of these rates by estimating the thermodynamic favorability of the pre- and post- cleavage secondary structures of the SCS. However, it is important to note some potential shortcomings of the thermodynamic approach to cascade design. Although the results of many of our experiments correlated with predictions, deviations from expected results occurred frequently enough to suggest that there are additional effects not accounted for by this approach. First, NUPACK calculates all binding interactions at thermodynamic equilibrium [1], [2], [78]. As upstream DNAzyme binding, cleavage, activator folding, and DNAzyme displacement are all dynamic processes; it was difficult at times to assess the relation between equilibrium predictions and dynamic circuit responses. This is particularly relevant for DNAzyme-catalyzed cleavage of SCS molecules where the DNAzyme does not need to form a complex that is stable in equilibrium but only needs to remain bound long enough to hydrolyze the RNA base at the cleavage site. Second, while looking at relative binding rates between SCS or ACT and downstream inhibitor was beneficial, the NUPACK predictions do not take into account the fact that the downstream DNAzyme and inhibitor are initially in a pre-formed complex. This can introduce error in the predictions because complementary sections of the downstream DNAzyme and inhibitor are normally unavailable for binding, leaving just the toehold single-stranded. This may have resulted in some of the aforementioned deviations between prediction and observed behavior. Third, even when sequences appeared to fold correctly, certain sequence motifs (particularly the presence of A-T pairs at the ends of stems) resulted in weakened structures or suggested that isoforms could be present with significant probabilities. Interestingly, in sequence selection for the final SCS design, the NUPACK design algorithm [72] routinely chose G-C rich outer stems and A-T rich inner stems. This suggests that the optimization algorithm was utilizing the higher bond strength of G-C base pairs to produce a maximally stable pre-cleavage structure and a minimally stable post-cleavage structure following removal of the outer stem as a cleavage product. This is a reasonable approach to sequence design for achieving our desired performance characteristics in the final SCS designs.

A potential alternative approach to SCS modeling would be to employ base-level coarse-grained dynamic simulations, as exemplified by the oxDNA software [25], [79], [80]. This may enable the estimation of rate constants for intermediate steps in the reaction mechanism. This is an important capability because certain small changes to the sequence and structure of the SCS designs were observed to have significant effects on performance.
